# PD-(L)1 inhibitors plus bevacizumab and chemotherapy as first-line therapy in PD-L1-negative metastatic lung adenocarcinoma: a real-world data

**DOI:** 10.1007/s00432-024-05637-1

**Published:** 2024-03-18

**Authors:** Yihui Ge, Yujing Zhan, Jie He, Juan Li, Jian Wang, Xiaojuan Wei, Chunni Wang, Aiqin Gao, Yuping Sun

**Affiliations:** 1 Phase I Clinical Research Center, Shandong University Cancer Center, Jinan, Shandong China; 2 Department of Radiation Oncology, Shandong Cancer Hospital and Institute, Shandong First Medical University and Shandong Academy of Medical Sciences, Jinan, China; 3 Department of Oncology, People’s Hospital of Zhangqiu District Jinan 250299, Shandong, People’s Republic of China; 4 Phase I Clinical Research Center, Shandong Cancer Hospital and Institute, Shandong First Medical University and Shandong Academy of Medical Sciences, Jinan, China; 5 Department of Medical Oncology, Qilu Hospital of Shandong University, Jinan, China; 6 Department of Oncology, The Affiliated Hospital of Qingdao University, Qingdao, 266003 Shandong Province China; 7 Department of Thoracic Radiation Oncology, Shandong Cancer Hospital and Institute, Shandong First Medical University & Shandong Academy of Medical Sciences, Jinan, Shandong China

**Keywords:** First-line therapy, PD-L1 negative, Lung adenocarcinoma, Efficacy

## Abstract

**Background:**

Chemotherapy combined with immune checkpoint inhibitors (IC), bevacizumab (BC), or both (IBC) is the preferred first-line therapy for PD-L1-negative and oncogenic-driver wild-type metastatic lung adenocarcinoma. However, the optimal strategy is still undetermined.

**Methods:**

This retrospective study enrolled PD-L1-negative metastatic lung adenocarcinoma patients from four cancer centers between January 1, 2018 and June 30, 2022. All the patients received IC, BC, or IBC as the first-line therapies. The efficacy and safety were evaluated.

**Results:**

A total of 205 patients were included, with 60, 83, and 62 patients in IC, BC, and IBC groups, respectively. The baseline characteristics among three groups were well balanced. Patients treated with IBC had the highest objective response rate (ORR) (43.5%) and disease control rate (DCR) (100%) relative to those treated with IC (40.4%, 84.2%) or BC (40.5%, 96.2%) (ORR: *P* = *0.919,* DCR: *P* < *0.01*). Compared with the IC (6.74 m) or BC (8.28 m), IBC treatment significantly improved median progression-free survival (mPFS) (9.53 m, *P* = *0.005*). However, no difference in overall survival (OS) was observed. When stratified by different clinical and molecular information, we found that male gender, ever smoking, wild-type genes mutations, and adrenal metastasis predict superior PFS benefit when treated with IBC. In patients with liver metastasis, IBC or BC treatment displayed better PFS compared with IC. No additional adverse reactions were observed in IBC group compared with other two groups.

**Conclusion:**

Combined IBC treatment achieved superior DCR and PFS compared with IC or BC in patients with PD-L1-negative metastatic lung adenocarcinoma, while did not increase the adverse events.

**Supplementary Information:**

The online version contains supplementary material available at 10.1007/s00432-024-05637-1.

## Introduction

Lung cancer is the second most prevalent and the most fatal tumor globally, with an increasing annual incidence rate (Sung et al. 2021). Lung adenocarcinoma is the predominant pathological type of lung cancer, accounting for more than half of all cases (Duma et al. 2019; Yao et al. 2021; Zhang et al. 2023). The majority of lung cancer patients are diagnosed at advanced stages, with a 5-year survival rate of merely 21–23% (Siegel et al. 2023).

Bevacizumab combined with chemotherapy (BC) was established as a standard first-line therapy for metastatic lung adenocarcinoma patients since 2006 (Zhou et al. 2015). Recently, immunotherapy using PD-1/PD-L1 inhibitors has greatly changed the paradigm of treatment. With the announcement of a series of clinical trials results including KEYNOTE-189, CameL, ORIENT-11, RATIONALE 304 and CHOICE-01, PD-1 inhibitors in combination with chemotherapy (IC) have become the preferred first-line therapy for lung adenocarcinoma (Ettinger et al. 2021; Hendriks et al. 2023; Owen et al. 2023; Garassino et al. 2023; Zhou et al. 2021a; Zhang et al. 2022; Lu et al. 2021; Wang et al. 2023). In addition, results from IMpower150 demonstrated the promising efficacy of combined bevacizumab, PD-L1 inhibitors, and chemotherapy (IBC) in the first-line setting, and provided a novel treatment mode (Socinski et al. 2018). Meanwhile, PD-L1 expression levels were identified as the most predictive biomarker for immune checkpoint inhibitors (ICIs) efficacy and used to select the optimal first-line schemes. It is reported that patients with high PD-L1 expression benefit more from ICIs, supporting that ICI-containing schemes should be given priority in these subpopulations. However, half of NSCLC patients were PD-L1 negative (Dietel et al. 2019). For these patients, the optimal scheme is still undetermined, especially in the real-world.

Here we performed this retrospective study to evaluate the first-line efficacy and safety of IBC, IC, and BC in PD-L1-negative metastatic lung adenocarcinoma in a real-world setting. Our findings will provide evidence for clinical decision-making for this subpopulation.

## Methods

This retrospective study enrolled 205 patients from 4 cancer centers (Shandong Cancer Hospital and Institute, QiLu Hospital of Shandong University, Shandong Provincial Hospital, and Affiliated Hospital of Qingdao University) from January 1, 2018, to June 30, 2022. The inclusion criteria were as follows: (1) lung adenocarcinoma confirmed by cytology or histology; (2) stage IV disease according to the American Joint Committee on Cancer Staging manual v8; (3) PD-L1 expression < 1%, as assessed using the Dako 22 C3 pharmDx test kit, and (4) receiving IBC, BC, or IC as first-line therapy. Patients harboring EGFR mutations and ALK/ROS-1 rearrangements were excluded. The last follow-up appointment was on July 19, 2023.

### Data collection and tumor response assessment

The demographic and clinicopathological information for all patients were recorded, which include age, sex, smoking history, metastatic sites, gene mutations, PD-L1 expression, treatment regimens and duration, salvage radiotherapy, and adverse events. Tumor response and progression were assessed using the Response Evaluation Criteria in Solid Tumors v1.1, determined as complete response (CR), partial response (PR), stable disease (SD), and progressive disease (PD). At least one lesion could be evaluated. CR was considered that all target lesions are gone and the short axis of any pathological lymph node must decrease to < 10 mm. PR was defined that the sum of the maximum diameter of the tumor target lesions has decreased by ≥ 30%. PD was defined that the sum of the maximum diameters of the tumor target lesions increases more than 20%, or new lesions appear. SD was defined that the sum of the maximum diameters of the tumor target lesions does not decrease to PR, or increases without reaching PD. Overall response rate (ORR) was defined as the percentage of patients who achieved CR and PR in all patients evaluated. Disease control rate (DCR) was defined as the sum of CR, PR, and SD rates. PFS was defined as the period from the initial first-line treatment administration to the date of disease progression or death from any cause. OS was defined as the duration from the initial first-line treatment administration to death because of any cause. The post-progression survival time was defined as the duration starting from the first radiological progression to the last follow-up or the time of death.

### Treatment

Patients received 4–6 cycles of IBC, IC or BC treatment at the induction stage. The platinum-based chemotherapy schemes include paclitaxel plus cisplatinum/carboplatin, or pemetrexed plus cisplatinum/carboplatin. Following the induction phase, patients receiving IBC or BC continued bevacizumab until unmanageable toxicity or disease progression, and patients receiving IBC or IC continued PD-(L)1 inhibitors until loss of clinical benefit. PD-(L)1 inhibitors included pembrolizumab, atezolizumab, sintilimab, camrelizumab, and triplimab.

### Toxicity evaluation

Adverse events were evaluated according to the National Cancer Institute Common Terminology Criteria for Adverse Events, v5.0. The evaluation criteria are displayed in supplement Table.

### Statistical analysis

SPSS v25.0 and GraphPad Prism 8 were used for statistical analyses. Descriptive statistics using counts (percentages) for categorical variables or medians for continuous variables were used to summarize the baseline characteristics of the patients. For categorical variables, Chi-square test or Fisher’s exact test was used to compare categorical variables. For continuous variables, one-way ANOVA test was conducted. We also used the Kaplan–Meier survival curve and log-rank test to compare the survival difference between two treatment groups. The Cox proportional hazards regression model was applied to assess the hazard ratio (HR) and corresponding 95% CI. *P* < *0.05* was considered statistically significant. Considering multiple testing, the Benjamini/Hochberg (B/H) was employed to regulate two-sided *p* values to control the false discovery rate (FDR). A correlation was deemed to be statistically significant, if its *p* value was < 0.05, corresponding to an FDR of 5%.

## Results

### Baseline clinical characteristics

A total of 205 patients were enrolled in this study—62 in the IBC group, 83 in the BC group, and 60 in the IC group. The median age for all patients was 60 years (range: 31–79). The proportion of female was 31.7%. 46.8% of patients had smoking history. The baseline characteristics of the three groups were broadly balanced. A detailed summary of the clinical characteristics of patients is shown in Table 1.

**Table 1:** Baseline clinical characteristics of all enrolled patients

Characteristic	IBC (*n* = 62)	BC (*n* = 83)	IC (*n* = 60)	*p* value
Median age (range)—yr	59(34–79)	60(31–79)	60.5(34–77)	0.703
Female sex—no. (%)	25(40.3)	24(28.9)	16(26.7)	0.209
Smoking no. (%)	25(40.3)	39(47.0)	32(53.3)	0.354
Distant metastasis no. (%)				
Brain	17(27.4)	20(24.1)	19(31.7)	0.609
Liver	10(16.1)	11(13.3)	7(11.7)	0.768
Bone	22(35.5)	35(42.2)	32(53.3)	0.134
Lung	29(46.8)	34(41.0)	22(36.7)	0.527
Pleura	15(24.2)	24(28.9)	11(18.3)	0.351
Adrenal	9(14.5)	15(18.1)	6(10.0)	0.407
Lymph node	48(77.4)	66(79.5)	51(85.0)	0.553
Gene mutation no. (%)				
No	28(45.2)	41(49.4)	33(55.0)	0.556
KRAS	13(21.0)	17(20.5)	13(21.7)	0.986
TP53	12(19.4)	12(14.5)	10(16.7)	0.738
Radiotherapy no. (%)	22(35.5)	34(41.0)	32(53.3)	0.125

### Tumor response to different treatment

Of 62 patients in the IBC group, 27 achieved PR and 35 SD, with an ORR of 43.5% and a DCR of 100%. In BC group, 32 patients obtained PR, 44 SD, and 3 PD. The ORR and DCR were 40.5% and 96.2%, respectively. Of 60 patients receiving IC treatment, 23 patients attained PR, 25 SD, and 9 PD. ORR and DCR for IC group were 40.4% and 84.2%, respectively. Patients receiving BC (*P* = *0.015*) or IBC (*P* = *0.001*) treatment showed the significantly improved DCR compared with those receiving IC. In addition, the IBC group had the numerically highest ORR among three groups although no significant difference was discovered (*p* = *0.919*). Detailed information is presented in Table 2.

**Table 2:** Efficacy of different treatments

Efficacy *n* (%)	IBC (*n* = 62)	BC (*n* = 83)	IC (*n* = 60)	*p* value
PR	27(43.5)	32(40.5)	23(40.4)	
SD	35(56.5)	44(55.7)	25(43.8)	
PD		3(3.8)	9(15.8)	
Not evaluated		4(4.8)	3(5.0)	
ORR	27(43.5)	32(40.5)	23(40.4)	0.919
DCR	62(100)	76(96.2)	48(84.2)	< 0.01
DCR	62(100)		48(84.2)	0.001
DCR		76(96.2)	48(84.2)	0.015

*PR* partial response; *SD* stable disease; *PD* progressive disease; *ORR* the percentage of patients who achieved CR and PR; *DCR* the sum of CR, PR, and SD rates

### Survival analyses based on different treatment

**Fig. 1:**
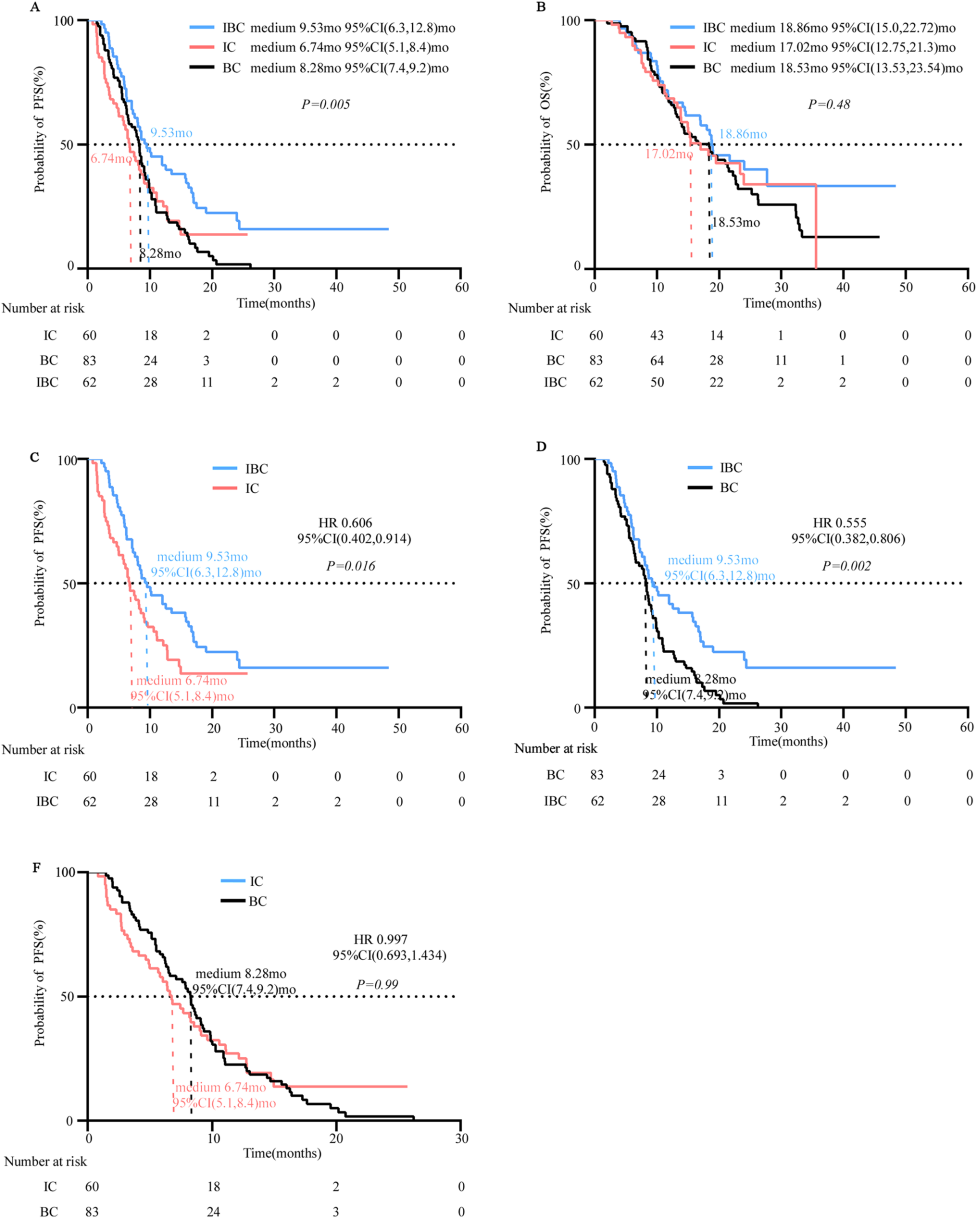
Progression-free survival and Overall survival in enrolled patients

Patients receiving IBC treatment displayed the longest PFS among three groups (*p* = *0.005*) (Fig. 1A). However, the medium OS were not significantly different (mOS: IBC vs. IC vs. BC:18.86 months vs. 17.02 months vs. 18.53 months; *p* = 0.48) (Fig. 1B). Then we conducted a pairwise comparison among three groups. We found that IBC treatment obtained a superior PFS benefit compared with either IC (medium: 9.53 months vs. 6.74 months; HR: 0.606; Fig. 1C) or BC treatment (medium: 9.53 months vs. 8.28 months; HR: 0.555; Fig. 1D). However, no PFS difference was observed between BC and IC groups (Fig. 1E).

### Survival in different subgroups

**Fig. 2:**
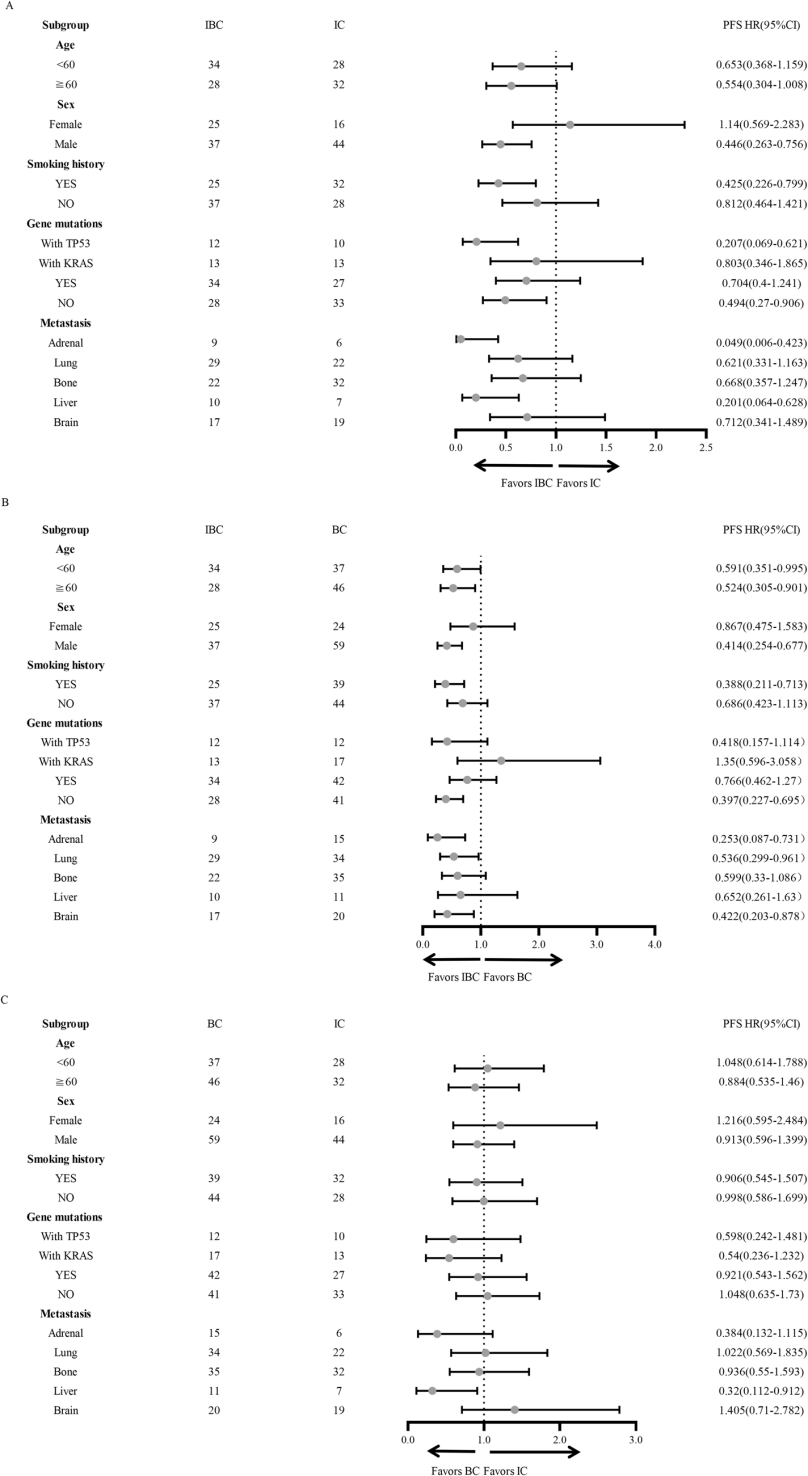
Forest plot of Subgroup analyses of Progression-free survival in enrolled patients

**Fig. 3:**
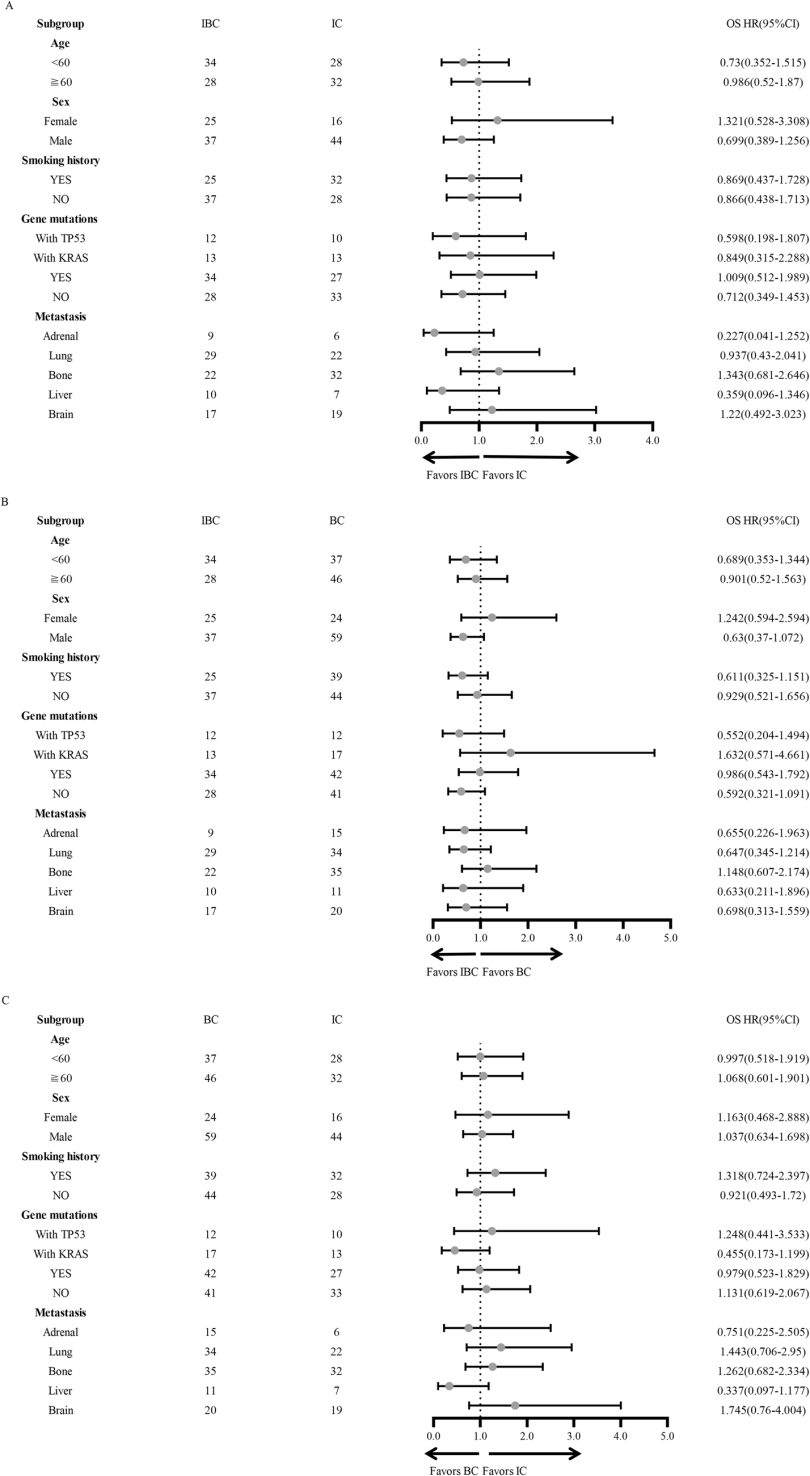
Forest plot of Subgroup analyses of Overall survival in enrolled patients

To further explore the features labeling potential beneficiary population, we conducted subgroup analyses based on different clinical characteristics including age, sex, smoking history, gene mutation subtypes, and different metastatic sites. We found patients with male gender, smoking history, TP53 mutation, wild-type genes, liver or adrenal metastases were more likely to obtain PFS benefit from IBC treatment compared with IC treatment (Fig. 2A). In addition, compared with BC, IBC treatment showed beneficial PFS in patients with male gender, smoking history, wild-type genes, adrenal metastasis, lung metastasis, and brain metastasis (Fig. 2B). However, compared with IC, BC treatment showed PFS benefit only in patients with liver metastasis (Fig. 2C). In terms of OS, no significant difference was observed in either subgroup among three treatments (Fig. 3A–C). However, we found that IBC treatment displayed a tendency of OS benefit compared with BC in male and in patients without gene mutations (Fig. 3B).

### The variations of first-line treatments and follow-up treatments

We collected the variations of first-line treatments and follow-up treatments due to the absence of difference in overall survival. In the first-line therapies, treatment discontinuation in IBC group (21.0%) was more common than those in BC (6.0%) and IC (11.7%) groups (*p* = *0.024*). At data cutoff, 171 patients achieved progression in total: 47 in IBC group, 76 in BC group, and 48 in IC group. The follow-up treatments included immunotherapy, anti-angiogenic therapy, TKI, and chemotherapy. We observed higher frequency of patients receiving immunotherapy (46%) or TKIs (9.2%) in BC group than those in IC (37.5%, 4.2%) and IBC (34%, 4.3%) groups. More importantly, patients who received immunotherapy in later lines showed numerically superior post-progression survival than those not (10.28 month vs. 6.89 month). In addition, we found more patients received ≥ 3rd-line treatment in BC (6.6%) and IC (8.3%) groups compared with IBC (4.3%) group. All information is summarized in Table 3.

**Table 3:** The variations of first-line treatments and follow-up treatments after progression

	IBC	BC	IC	*p* value
The variations of first-line treatments (*n*)	*n* = 62	*n* = 83	*n* = 60	
Dose reduction no. (%)	2(3.2%)	2(2.4%)	1(1.7%)	0.854
Treatment discontinuation no. (%)	13(21%)	5(6.0%)	7(11.7%)	0.024
Follow-up treatments (*n*)	*n* = 47	*n* = 76	*n* = 48	
Immunotherapy no. (%)	16(34.0%)	35(46.0%)	18(37.5%)	0.374
Anti-angiogenic therapy no. (%)	12(25.5%)	4(5.3%)	11(22.9%)	0.003
TKI no. (%)	2(4.3%)	7(9.2%)	2(4.2%)	0.417
Chemotherapy no. (%)	26(55.3%)	35(46.0%)	26(54.2%)	0.526
Unknow no. (%)	4(8.5%)	10(13.2%)	6(12.5%)	0.723
No no. (%)	11(23.4%)	17(22.4%)	9(18.8%)	0.841
Lines of follow-up treatments (*n*)				
< 3 no. (%)	45(95.7%)	71(93.4%)	44(91.7%)	0.710
≥ 3 no. (%)	2(4.3%)	5(6.6%)	4(8.3%)	

Immunotherapy: immune checkpoint inhibitors alone or in combined with chemotherapy ± anti-angiogenic inhibitors; anti-angiogenic therapy: anti-angiogenic inhibitors ± chemotherapy; TKI: tyrosine kinase inhibitors

### Safety

In generally, the occurrence of adverse events in the three groups is comparable, with a total rate of 45%, 39.8%, and 30.6% (*p* = *0.254*), and grade III–IV adverse events of 13.3%, 20.4%, and 11.3% in IC, BC, and IBC groups, respectively (*p* = *0.271*). Transaminases increase was the most frequent grade I–II adverse event in IC treatment group (23.3%), while the most frequent event was nausea/vomiting in IBC (29.0%) and BC (25.3%) groups. Neutropenia was the predominant grade III–IV adverse event across the three groups. The IBC treatment commonly led to grade I–II nausea/vomiting and grade III–IV neutropenia (8.1%), similar to those in the BC (11.0%) and IC (5.0%) groups. Moreover, the ratio of grade I–II dermal toxicity (6.7%) and pneumonitis (10.0%) in the IC group was higher than that in the IBC group, whereas the frequency of grade I–II hypothyroidism (3.2%) and RCCEP (reactive cutaneous capillary endothelial proliferation) (6.4%) in the IBC group was higher than that in the IC group. Moreover, infusion-related reactions occurred only in the IBC group. Grade I–II bleeding was observed in the BC group alone. All adverse events are listed in Table 4.

**Table 4:** Adverse events based on different treatment options

Adverse events, no.(%)	IBC (*n* = 62)		BC (*n* = 83)		IC (*n* = 60)	
Any grade	19(30.6)		33(39.8)		27(45)	
Grade III-IV	7(11.3)		17(20.4)		8(13.3)	
Adverse events, no.(%)	Grades I–II	Grades III–IV	Grades I–II	Grades III–IV	Grades I–II	Grades III–IV
Transaminases increased	17(27.4)		19(22.8)	1(0.8)	14(23.3)	1(0.9)
Infusion-related reaction		1(1.6)				
Leukopenia	15(24.2)	4(6.6)	16(19.2)	7(8.4)	10(16.7)	3(5.0)
Neutropenia	14(22.6)	5(8.1)	16(19.2)	9(11.0)	9(15.0)	3(5.0)
Anemia	5(8.1)		9(10.8)	1(1.2)	9(15.0)	
Thrombocytopenia	6(9.7)	2(3.2)	7(8.4)	2(2.4)	3(5.0)	
Nausea/vomiting	18(29.0)		21(25.3)	4(4.8)	9(15.0)	
Diarrhea					1(0.9)	
Fatigue	1(1.6)		5(6.0)		2(3.2)	
Dermal toxicity	2(3.2)	1(1.6)	1(1.2)		4(6.7)	
Bleeding			2(2.4)			
Pneumonitis	2(3.2)	1(1.6)			6(10.0)	1(1.6)
Hypothyroidism	2(3.2)				1(1.6)	
RCCEP	4(6.4)					

*RCCEP* reactive cutaneous capillary endothelial proliferation

## Discussion

Lung adenocarcinoma represents the most common pathological subtype of NSCLC, accounting for 60% of all cases (Travis et al. 2015). In the pre-immunotherapy era, BC is considered as the preferred treatment for metastatic lung adenocarcinoma patients regardless of PD-L1 expression (Sandler et al. 2006; Hirsch et al. 2017). Since last decade, immunotherapy targeting PD-1/PD-L1 and their combination strategies including IC and IBC have revolutionized lung adenocarcinoma treatment, especially in patient with high PD-L1 expression (Li et al. 2023; Shao et al. 2022; Wenfan et al. 2023). However, the most beneficial strategy for PD-L1-negative patients is still undetermined.

There is still no prospective clinical trials directly comparing the efficacy of IBC, IC, and BC in PD-L1-negative patients. The biomarker subgroup analysis from the IMpower150 trial indicated that IBC treatment yield superior PFS but not OS compared with BC, while IC treatment generated similar OS compared with BC (Socinski et al. 2021). In addition, two small-sample retrospective studies compared the first-line efficacy of IC and BC in lung adenocarcinoma patients, but controversial conclusions were obtained (Xia et al. 2023; Huang et al. 2022). Huang et.al observed similar PFS and OS in IC and BC groups, while Xia et.al found BC has longer PFS and OS in PD-L1-negative NSCLC compared with IC. Moreover, the real-world efficacy of IBC was not explored by any of these studies. Here we are the first to compare the real-world efficacy of IBC, IC, and BC in PD-L1-negative NSCLC, aiming to explore the optimal strategy for this population and guild clinical decision-making. We found IBC treatment exhibited a significantly enhanced ORR and mPFS compared to IC or BC, but no OS benefit was observed. In addition, no difference in PFS and OS was observed between IC and BC groups. These data indicated that patients who have severe symptoms or high tumor burden should preferably choose IBC for first-line strategy due to better short-term response.

The synergistic effect of bevacizumab and ICIs has been evidenced by multiple preclinical studies. It is reported by normalizing tumor vasculature, bevacizumab can reinstate the pro-immunogenic conditions in the TME, characterized by increased intra-tumoral infiltration of cytotoxic T cells, decreased MDSCs, regulatory T cells and M2 macrophages (Fukumura et al. 2018). Based on the above theory, numerous clinical trials have conducted to demonstrate the potential of bevacizumab in improving ICI efficacy. The ORIENT-31 study shows that in patients with sensitive EGFR mutations but resistant to EGFR-TKIs, bevacizumab plus sintilimab and chemotherapy generated better PFS and ORR compared with chemoimmunotherapy or chemotherapy alone (Lu et al. 2022). Similarly, in the IMpower150 study, the ABCP group showed longer PFS and OS compared to the BCP group in ITT and PD-L1-positive population. However, in PD-L1-negative subgroup, these schemes showed comparable OS. Consistently, our real-world data showed that PD-(L)1 inhibitors plus bevacizumab and chemotherapy generated superior PFS rather than OS compared with IC or BC in PD-L1-negative patients, suggesting that bevacizumab might improve the suppressive tumor immune environment (TIME) in this immuno-resistant subpopulation. Further analysis of TIME in PD-L1-negative population is needed to verify our hypothesis.

To further identify beneficiary population from different treatment, we performed subgroup analysis of PFS and OS. We found that patients with male gender, smoking history, wild-type genes, and adrenal metastasis benefit more from IBC than from IC or BC. For patients with brain metastasis, IBC showed superior PFS than BC treatment. In addition, patients with liver metastasis obtained better PFS from bevacizumab-containing treatment (IBC and BC) than from IC. It is reported that male patients tend to have higher levels of CD8^+^T cells and tumor mutational burden (TMB), which predicts better response to immunotherapy (Leun et al. 2020; Klein and Flanagan 2016). Furthermore, patients with smoking history and wild-type genes are reported to have higher TMB (Li et al. 2020). We speculate that improvement of suppressive TIME by bevacizumab plays more obvious role in these subpopulation compared with that in their counterparts. It is reported that brain and liver metastasis lesions displayed an immunosuppressive state due to lack of tumor-infiltrating lymphocytes (TILs) [Li et al. 2022; Deng et al. 2023]. In addition, high VEGF level in the liver metastasis promotes tumor angiogenesis and leads to immune evasion by restricting the maturation of DCs and by reducing the expression of selectins, integrins, and adhesion molecules (Reck et al. 2023). Combination therapy with immune checkpoint inhibitors and anti-angiogenic inhibitors can synergically block these pathways, reprogram the unfavorable TIME and enhance immunotherapy efficacy (Zhou et al. 2021b).

Unfortunately, we did not observe prolonged OS by IBC treatment despite significantly improved PFS. In our effort to explain the potential probability, we analyzed subsequent treatments and first-line variation including discontinuation and dose reduction. We found IBC group had higher frequency of treatment discontinuation than other groups, which might impair OS benefit. We also found more patients in BC group received immunotherapy in later lines, which is associated with prolonged post-progression survival in our and other studies (Long et al. 2017; Guven et al. 2023). In addition, more patients achieved ≥ 3rd-line treatment in BC and IC group compared with IBC group numerically. But no statistical significance was observed due to the high incidence of loss to follow-up in real-world probably. These data could partly explain the similar OS among three groups.

Our study had some limitations. First, the number of patients included in each treatment group is limited. Second, as this is a retrospective study, patients cannot be perfectly matched. Therefore, our results need further confirmation from prospective clinical trials.

## Conclusion

Combined IBC treatment achieved superior DCR and PFS compared with IC or BC treatment in patients with PD-L1-negative metastatic lung adenocarcinoma, but did not increase the adverse events.

## Supplementary Information

Below is the link to the electronic supplementary material.

Supplementary file1 (DOCX 17 KB)

## Data Availability

The data are not publicly available due to [restrictions e.g., their containing information that could compromise the privacy of research participants].
