# Supplementary material for: PD-(L)1 inhibitors plus bevacizumab and chemotherapy as first-line therapy in PD-L1-negative metastatic lung adenocarcinoma: a real-world data
Source: J Cancer Res Clin Oncol. 2024 Mar 18;150(3):135. doi: 10.1007/s00432-024-05637-1 (PMC10948463; doi:10.1007/s00432-024-05637-1)
Supplement: Supplementary file 1 — Supplementary file1 (DOCX 17 KB) [file 432_2024_5637_MOESM1_ESM.docx]

**Supplement table. Adverse Events**

| Adverse Events,no.(%) | Grades I | Grades II | Grades III | Grades IV |
| --- | --- | --- | --- | --- |
| Transaminases Increased:Including Aspartate aminotransferase increased and Alanine aminotransferase increased | > ULN - 3.0 x ULN if baseline  was normal; 1.5 - 3.0 x  baseline if baseline was  abnormal | > 3.0 - 5.0 x ULN if baseline  was normal; 3.0 - 5.0 x  baseline if baseline was  abnormal | > 5.0 - 20.0 x ULN if baseline  was normal; 5.0 - 20.0 x  baseline if baseline was  abnormal | > 20.0 x ULN if baseline  was normal; 20.0 x  baseline if baseline was  abnormal |
| Infusion-related Reaction | Mild transient reaction;  infusion interruption not  indicated; intervention not  indicated | Therapy or infusion  interruption indicated but  responds promptly to  symptomatic treatment(e.g., antihistamines,NSAIDS,  narcotics, IV fluids);  prophylactic medications  indicated for <=24 hrs | Prolonged (e.g., not rapidly  responsive to symptomatic  medication and/or brief  interruption of infusion);  recurrence of symptoms  following initial improvement;  hospitalization indicated for  clinical sequelae | Life-threatening  consequences; urgent  intervention indicated |
| Leukopenia | <LLN - 3000/mm3; <LLN - 3.0 x  10e9 /L | <3000 - 2000/mm3; <3.0 - 2.0  x 10e9 /L | <2000 - 1000/mm3; <2.0 - 1.0  x 10e9 /L | <1000/mm3; <1.0 x 10e9 /L |
| Neutropenia | <LLN - 1500/mm3; <LLN - 1.5 x  10e9 /L | <1500 - 1000/mm3; <1.5 - 1.0  x 10e9 /L | <1000 - 500/mm3; <1.0 - 0.5 x  10e9 /L | <500/mm3; <0.5 x 10e9 /L |
| Anemia | Hemoglobin (Hgb) <LLN - 10.0  g/dL; <LLN - 6.2 mmol/L; <LLN  - 100 g/L | Hgb <10.0 - 8.0 g/dL; <6.2 - 4.9  mmol/L; <100 - 80g/L | Hgb <8.0 g/dL; <4.9 mmol/L;  <80 g/L; transfusion indicated | Life-threatening  consequences; urgent  intervention indicated |
| Thrombocytopenia | <LLN - 75,000/mm3; <LLN -  75.0 x 10e9 /L | <75,000 - 50,000/mm3; <75.0  - 50.0 x 10e9 /L | <50,000 - 25,000/mm3; <50.0  - 25.0 x 10e9 /L | <25,000/mm3; <25.0 x 10e9 /L |
| Nausea/Vomiting | Loss of appetite without  alteration in eating habits | Oral intake decreased without  significant weight loss,  dehydration or malnutrition | Inadequate oral caloric or  fluid intake; tube feeding,  TPN, or hospitalization  indicated |  |
| Diarrhea | Increase of <4 stools per day  over baseline; mild increase in  ostomy output compared to  baseline | Increase of 4 - 6 stools per day  over baseline; moderate  increase in ostomy output  compared to baseline; limiting  instrumental ADL | Increase of >=7 stools per day  over baseline; hospitalization  indicated; severe increase in  ostomy output compared to  baseline; limiting self care ADL | Life-threatening  consequences; urgent  intervention indicated |
| Fatigue | Fatigue relieved by rest | Fatigue not relieved by rest;  limiting instrumental ADL | Fatigue not relieved by rest,  limiting self care ADL |  |
| Dermal Toxicity | <10% BSA, which  may or may not be associated  with symptoms of pruritus or  tenderness | covering 10 - 30% BSA, which  may or may not be associated  with symptoms of pruritus or  tenderness; associated with  psychosocial impact; limiting  instrumental ADL; covering >  30% BSA with or without mild  symptoms | >30% BSA with  moderate or severe  symptoms; limiting self-care  ADL; associated with local  superinfection with oral  antibiotics indicated | Life-threatening  consequences; covering any % BSA,  which may or may not be  associated with symptoms of  pruritus or tenderness and are  associated with extensive  superinfection with IV  antibiotics indicated |
| Bleeding | Mild symptoms; intervention  not indicated | Moderate symptoms;  intervention indicated | Transfusion indicated;  invasive intervention  indicated; hospitalization | Life-threatening  consequences; urgent  intervention indicated |
| Pneumonitis | Asymptomatic; clinical or  diagnostic observations only;  intervention not indicated | Symptomatic; medical  intervention indicated;  limiting instrumental ADL | Severe symptoms; limiting self  care ADL; oxygen indicated | Life-threatening respiratory  compromise; urgent  intervention indicated (e.g.,  tracheotomy or intubation |
| Hypothyroidism | Asymptomatic; clinical or  diagnostic observations only;  intervention not indicated | Symptomatic; thyroid  replacement indicated;  limiting instrumental ADL | Severe symptoms; limiting self  care ADL; hospitalization  indicated | Life-threatening  consequences; urgent  intervention indicated |
| RCCEP | <10% BSA, which  may or may not be associated  with symptoms of pruritus or  tenderness | covering 10 - 30% BSA, which  may or may not be associated  with symptoms of pruritus or  tenderness; associated with  psychosocial impact; limiting  instrumental ADL; covering >  30% BSA with or without mild  symptoms | >30% BSA with  moderate or severe  symptoms; limiting self-care  ADL; associated with local  superinfection with oral  antibiotics indicated | Life-threatening  consequences; covering any % BSA,  which may or may not be  associated with symptoms of  pruritus or tenderness and are  associated with extensive  superinfection with IV  antibiotics indicated |

RCCEP: reactive cutaneous capillary endothelial proliferation.
